# Effect of Sunflower Lecithin Supplementation on Meibomian Gland Function in Adults With Dry Eye Disease

**DOI:** 10.7759/cureus.98738

**Published:** 2025-12-08

**Authors:** Sinan Akosman, Sai Samayamanthula, Arnold Leigh, Renxi Li, Maya Bitar, Keith Wroblewski

**Affiliations:** 1 Ophthalmology, George Washington University School of Medicine and Health Sciences, Washington, D.C., USA; 2 Ophthalmology/Orthopaedic Surgery, University of Virginia School of Medicine, Charlottesville, USA; 3 Clinical and Translational Research, George Washington University School of Medicine and Health Sciences, Washington, D.C., USA

**Keywords:** dry eye disease, lecithin supplementation, meibomian gland dysfunction, prospective randomized clinical trial, sunflower lecithin

## Abstract

Background: Dry eye disease (DED), often associated with meibomian gland dysfunction (MGD), affects millions globally, yet effective treatments remain limited. Sunflower lecithin, rich in phosphatidylcholine, may enhance lipid secretions from the meibomian glands, potentially stabilizing the tear film and reducing evaporative loss. We tested the hypothesis that sunflower lecithin supplementation can improve the signs and symptoms of DED in adults with MGD.

Methods: In this double-blind, single-center clinical trial, 31 patients with DED were randomized in a 2:1 ratio to receive either sunflower lecithin (4,800 mg/day) or placebo (4,000 mg olive oil/day) for 90 days. At baseline and the end of the study, DED symptoms were evaluated using the Standard Patient Evaluation of Eye Dryness (SPEED) questionnaire. DED signs were evaluated through tear film breakup time (TBUT), meibomian gland score (MGS), and ocular surface inflammation, measured via matrix metalloproteinase-9 (MMP-9) detection using the InflammaDry test (QuidelOrtho, San Diego, USA). For both groups, mean changes were calculated, and unpaired t-tests were used to compare mean values between groups with statistical significance set at P<0.05. Additionally, the mean difference between the active and placebo groups was calculated for each outcome to quantify the magnitude of improvement or worsening in the active group relative to placebo.

Results: Active and placebo groups showed decreases in SPEED scores with mean changes of -3.21 ± 1.47 and -1.50 ± 3.34 points, respectively, resulting in a mean difference of -1.71 (95% CI: -5.36 to 1.94; P=0.29). TBUT, MGS, and InflammaDry results improved in both groups but showed no significant differences between groups. Both groups exhibited improvements across all metrics with no significant differences.

Conclusion: Sunflower lecithin supplementation did not demonstrate a significant therapeutic benefit over placebo in improving MGD-related DED signs or symptoms. Further research with larger sample sizes, longer follow-up, and combination therapies is recommended to explore the potential role of phospholipids in DED management.

## Introduction

Dry eye syndrome (DES), also known as keratoconjunctivitis sicca (KCS), is a common reason for eye doctor visits. DES is a multifactorial disease of the ocular surface, characterized by a loss of homeostasis of the tear film and accompanied by symptoms of eye discomfort, visual disturbance, and tear film instability. The tear film, approximately 2-5.5 μm thick, consists of three layers: lipid, aqueous, and mucin. Disruption in any of these components can lead to dry eye disease (DED) [1].

Although the pathogenesis of DED is not fully understood, it is primarily recognized as an inflammatory condition, involving proinflammatory cytokines, chemokines, matrix metalloproteinases, and autoreactive T-helper cells. DES can be classified into two types: aqueous-deficient, characterized by reduced tear production, and hyper-evaporative, characterized by increased tear film evaporation. The hyper-evaporative type is thought to be the most prevalent etiology and is often associated with meibomian gland dysfunction (MGD), which is the leading cause of DES [2].

The meibomian glands are large sebaceous glands located along the upper and lower eyelid margins. These glands secrete meibum, a substance that forms the superficial lipid layer of the ocular tear film. The lipid contributions from the meibomian glands stabilize the tear film and protect against the evaporation of the aqueous phase [3]. MGD is a largely underdiagnosed and undertreated condition that involves various dysfunctions in the quality and quantity of meibomian gland secretions. It is characterized by chronic inflammation of the meibomian glands, leading to terminal duct obstruction and/or qualitative or quantitative changes in glandular secretion. These changes can disrupt the tear film and cause symptoms of eye irritation and inflammation of the ocular surface. Treatment for MGD is mainly preventative, including measures such as warm compresses and regular eyelid massages [4].

Lecithins are a group of essential phospholipids involved in various biological functions in the human body, including lipid-cholesterol transport, transmembrane signaling, and neurological function [5]. Dietary lecithin supplementation has been studied in breastfeeding mothers for its role as an emulsifier, helping alleviate plugged milk ducts by reducing ductal fat deposits, which can cause painful inflammation [6,7]. Sunflower lecithin supplements are also rich in phosphatidylcholine [8], an essential nutrient for maintaining ocular function [9]. Choline deficiencies have been linked to various ocular conditions, including retinal diseases, glaucoma, DES, and disorders of the lens, optic nerve, and visual cortex. As a precursor of acetylcholine, choline stimulates secretions from the lacrimal glands through the parasympathetic nervous system, and its deficiency may reduce parasympathetic tone, contributing to DES pathophysiology. Additionally, choline is thought to have anti-inflammatory properties, which may play a role in the tissue inflammation that is a hallmark of DES [10]. Choline supplementation has been found to suppress tissue inflammation and oxidative damage and promote wound healing on the ocular surface by inhibiting pro-inflammatory cytokines. Lastly, phosphatidylcholine itself is a key component of the lipid layer of the tear film, which is responsible for delaying tear evaporation. Given these various implications of lecithins in the natural functioning of the ocular surface and physiology, we hypothesized that choline supplementation may enhance lipid secretions by the meibomian glands, thereby improving tear film stability and reducing evaporative tear loss in patients with dry eyes [11]. This study was conducted to evaluate the effect of oral sunflower lecithin on the functioning of meibomian glands in adults with DES.

The primary outcome in this study was the Standard Patient Evaluation of Eye Dryness (SPEED) questionnaire difference over a 90‑day period to evaluate the patient’s own subjective perception of improvement in symptoms. The secondary objectives assessed clinical signs of supplementation through the tear film break-up time (TBUT), meibomian gland score (MGS), and ocular surface inflammation assessments.

## Materials and methods

Trial population

From May 30, 2023, to August 2, 2023, a total of 85 patients with a clinical diagnosis of DED were screened at the George Washington Medical Faculty Associates (GW MFA) Ophthalmology Clinic, Washington, D.C. Eligibility criteria included age greater than 18 years, a diagnosis of DES in both eyes, and the presence of lid margin abnormalities such as telangiectasia or meibomian gland plugging as determined by slit-lamp examination. In addition, patients were required to meet specific signs of tear film dysfunction: a fluorescein TBUT (the time from a blink to the appearance of gaps in the tear film, with shorter times indicating greater abnormality) of 10 seconds or less in each eye, best-corrected visual acuity (BCVA) of 20/40 or better in each eye, and a SPEED questionnaire score greater than six out of a total of 28 points.

Patients were excluded if they had any pre-existing ocular conditions other than DED, were unable to swallow soft-gel capsules, or had undergone significant dietary changes or begun using supplements in the month preceding the study. Other exclusion criteria included pregnancy, breastfeeding, current smoking status, the use of anticholinergic drugs, and ocular surgery within the past six months. Patients taking other fatty acid supplements were also excluded.

Of the 85 patients screened, 31 (36%) were eligible for inclusion in the trial. The protocol was approved by the George Washington University Committee on Human Research Institutional Review Board on April 26, 2023, and the study was registered at ClinicalTrials.gov (Registration Number: NCT06058559). All the patients provided written informed consent.

Inclusion and exclusion criteria 

Inclusion criteria for the study included age greater than 18 at the time of informed consent, clinical diagnosis of dry eye in both eyes, fluorescein TBUT less than 10 seconds in both eyes, the presence of lid margin scaling, telangiectasia, collarette or meibomian gland plugging on slit-lamp examination, BCVA (Snellen) of 20/40 or better in each eye, SPEED questionnaire score >6 to <14, ability to understand, be willing and able, and to agree with study procedures, visit schedule, and restrictions.

Exclusion criteria included any pre-existing ocular disease other than DED, patient inability to swallow soft-gel capsules, severe illness, pregnancy or breastfeeding, smoking, and regular use of strongly anticholinergic drugs, drastic change of food and/or food supplements within the last month, and other food supplements with fatty acids. Ocular exclusion criteria also included evidence of acute ocular infection and/or intraocular inflammation within one month prior to the onset of this study, ocular surgery within the last six months, patients treated with topical ocular, steroidal, or nonsteroidal, anti-inflammatory treatment within the last month, occlusion therapy with lacrimal or punctum plugs within the last three months, alterations of the lacrimal drainage system, eyelid abnormalities, patients on oral tetracycline or corticosteroids, and active allergy or infection at the ocular surface.

Trial groups

Upon study enrollment, patients were randomly assigned in a 2:1 ratio to either the active or placebo group. Randomization was conducted using a computer-generated schedule to ensure unbiased assignment. The randomization schedule was prepared by an independent statistician, who was not involved in the conduct of the study. The active group received four sunflower lecithin soft-gel capsules (1,200 mg each) per day, while the placebo group received four olive oil capsules (1,000 mg each) per day. The supplementation regimen was followed for 90 days. Participants were responsible for the daily at-home administration of their assigned study product, either active sunflower lecithin or placebo. Lecithin is usually well tolerated and is "generally recognized as safe" (GRAS) by the U.S. Food and Drug Administration (FDA). Manufacturer guidelines and prior studies of lecithin supplementation recommend a daily dose of 4,800 mg, which was the dose used in this study [5-7].

Olive oil was selected as the placebo because of its neutral properties and safe profile. Each placebo capsule contained 1,000 mg of extra virgin olive oil, which is commonly used as a placebo in randomized clinical trials involving omega-3 fatty acid supplementation [12-18]. The daily quantity of olive oil administered in this study, approximately 4 g (or about 1 teaspoon), is not only considered safe but has also been associated with a reduced risk of cardiovascular disease [16]. This dosage is significantly lower than the amounts typically studied for health benefits in Mediterranean diet interventions, which often provide at least 60 g per day [16,19,20]. 

Participants were provided with the entire duration of lecithin capsules or placebo upon enrollment at the baseline visit. Both interventions were packaged into nondescript white packer bottles, with each participant receiving bottles containing a total of 360 soft-gel capsules (four capsules/day for 90 days) of either active ingredient or placebo. Participants had the autonomy to withdraw from the study at any time. The investigational products were virtually identical in appearance and dosage, with both active and placebo capsules dispensed in nondescript and unlabeled white bottles to maintain blinding of both the patients and the investigators.

Outcome measures

The primary outcome of the study was the change in dry eye symptoms, as determined by the SPEED questionnaire [21] between baseline and day 90. The SPEED questionnaire is a validated 0-28 point patient-reported outcome measure. Patients rated the frequency and severity of common dry eye symptoms, with higher scores indicating greater symptom burden. The primary outcome was the change in total SPEED score from baseline to day 90, with negative values reflecting symptomatic improvement.

Secondary outcomes included changes in TBUT and MGS [22] and matrix metalloproteinase-9 (MMP-9) positivity via the InflammaDry immunoassay (QuidelOrtho, San Diego, USA). 

TBUT

TBUT was measured utilizing the fluorescein dye strip with slit lamp examination. A sterile 1 mg fluorescein strip was moistened with nonpreserved saline and lightly applied to the inferior palpebral conjunctiva. Patients were instructed to blink several times to evenly distribute the dye. Using cobalt blue illumination, the patient blinked once and kept the eyelids open without blinking. TBUT was defined as the interval between the last blink and the first visible discontinuity in the fluorescein-stained tear film. A stopwatch was used for timing, and premature blinking would prompt a repeat measurement. Three consecutive TBUT measurements were obtained per eye, with brief pauses between trials. The mean of the three measurements was recorded as the TBUT for that eye.

MGS 

MGS was determined under slit lamp examination. For each eye, the lid margin appearance, gland expressibility, and a qualitative meibum score were evaluated on a scale ranging from 0 (best) to 3 (worst). For the lid margin score, one point was assigned for the presence of each of the following: lid margin irregularity, vascular engorgement, and glandular orifice obstruction. Gland expressibility of the five medial glands was scored as follows: 0 = all five glands, 1 = 3-4 glands, 2 = 1-2 glands, and 3 = no glands expressed. Meibum was scored as follows: 0 = clear, 1 = cloudy, 2 = cloudy with debris, 3 = inspissated. As such, a total of 18 points (nine for each eye) was scored for each participant.

InflammaDry Immunoassay

Ocular surface inflammation was assessed through MMP-9 detection using the InflammaDry immunoassay. The test involved collecting a tear sample from the patient’s palpebral conjunctiva by gently dabbing the area 6-8 times, allowing the patient to blink after every 2-3 dabs. Once the sampling fleece was saturated with tears (approximately 10 µL), it was assembled onto the test cassette. The test was activated by dipping the absorbent pad into a buffer solution for about 20 seconds, with results interpreted after 10 minutes. A positive test result, indicative of elevated MMP-9 levels (≥40 ng/mL), was marked by the appearance of one blue control line and one red line in the result window. A single blue control line indicated a negative test result (<40 ng/mL). In this study, a positive test result was denoted with a value of 1, and a negative test result with a value of 0.

The study comprised two visits: a combined screening and baseline visit (Visit 1, day 0) for patient recruitment, consent, and initial assessments, and an end-of-study visit (Visit 2, day 90) to evaluate changes following supplementation. During both visits, the SPEED questionnaire score, TBUT, MGS, and InflammaDry test were measured and recorded. All participants, clinical staff, and laboratory personnel were blinded to the trial-group assignments. Additionally, visual acuity and intraocular pressure (IOP) were measured as part of the study's safety assessments. Both MGS and SPEED are open-access clinical instruments and do not require a license for use in research or clinical practice.

Statistical analysis

For the primary and secondary outcomes, baseline values were taken as the mean of the values obtained during Visit 1. The values used to assess changes were calculated as the difference between the Visit 1 and Visit 2 values. The differences for both the active and placebo groups were then averaged to calculate the mean, and 95% confidence intervals (CIs) were calculated for these mean differences, as well as the standard deviation (SD) was calculated. Unpaired two-sample t-tests were performed to compare the mean values between the two groups and determine statistical significance. A two-sided P-value of <0.05 was considered statistically significant. In addition, the mean difference between the active and placebo groups was calculated for each outcome, along with the corresponding 95% CIs, to quantify the magnitude of improvement (or worsening) in the active group relative to the placebo group. Analyses were performed according to the as-treated principle.

Categorical outcomes, such as the InflammaDry assay, were analyzed as binary variables. Between-group differences in the proportion of positive versus negative results were summarized, with the change in positivity from baseline to day 90 recorded for each group.

Microsoft Excel (Microsoft Corp., Redmond, USA) was used to calculate means, SDs, between-group differences, 95% CIs, and to generate all graphical representations of the data.

## Results

Of the 31 patients who provided consent and were randomized, 11 withdrew from the study after consenting. The remaining 20 patients contributed data to the final analysis, with 14 in the active supplement group and six in the placebo group. In both groups, SPEED questionnaire scores decreased between the baseline and the end of the study. The mean (±SD) change in the SPEED score was -3.21 ± 1.47 points in the active supplement group and -1.50 ± 3.34 points in the placebo group, resulting in a mean difference in change of -1.71 points (95% CI: -5.36 to 1.94; P=0.29); TBUT increased by 3.40 ± 4.06 seconds as compared to 2.73 ± 5.01 seconds, with a mean difference in change of 0.67 seconds (95% CI: -5.78 to 7.12; P=0.85); mean (±SD) change in the MBS was -1.14 ± 2.08 as compared to -0.90 ± 3.57, with a mean difference of -0.24 points (95% CI: -4.37 to 3.89; P=0.91); and the change in the InflammaDry test results was -0.40 ± 0.48 as compared to -0.17 ± 0.22, with a mean difference of -0.23 (95% CI: -0.87 to 1.33; P=0.33). Overall, improvements were observed across all metrics in both groups; however, no statistically significant differences were found between the active supplement and placebo groups for any of the measured outcomes (Table 1).

**Table 1:** Primary and secondary outcomes Baseline values were the means of values obtained during the screening and eligibility visit (Visit 1). The values used for assessing change from baseline were the means of values obtained during the final visit (Visit 2). *SPEED questionnaire [21] scores range from 0 to 28, with a score of 0 indicating no ocular discomfort and higher scores indicating greater symptom severity. **TBUT is the time from a blink to the appearance of gaps in the tear film, with shorter times indicating greater abnormality. ***MGS was evaluated using a meibomian gland evaluator instrument (Dry Eye Rescue, Boca Raton, USA) via slit lamp [22]. For each eye, the lid margin appearance, gland expressibility, and a qualitative meibum score were evaluated on a scale ranging from 0 (best) to 3 (worst). For the lid margin score, one point was assigned for the presence of each of the following: lid margin irregularity, vascular engorgement, and glandular orifice obstruction. Gland expressibility of the five medial glands was scored as follows: 0 = all five glands, 1 = 3-4 glands, 2 = 1-2 glands, and 3 = no glands expressed. Meibum was scored as follows: 0 = clear, 1 = cloudy, 2 = cloudy with debris, 3 = inspissated. As such, a total of 18 points (nine for each eye) was scored for each participant. ****InflammaDry immunoassay was used to assess ocular surface inflammation through MMP-9 detection. A positive test result was denoted with a value of 1, and a negative test result with a value of 0. SPEED, Standard Patient Evaluation of Eye Dryness; TBUT, tear film break-up time; DED, dry eye disease; MGS, meibomian gland score; MMP-9, matrix metalloproteinase-9; SD, standard deviation; CI, confidence interval

Signs of DED	Active supplement (N=14)		Placebo supplement (N=6)		Mean difference (95% CI)	P-value
	No. of patients	Mean change (±SD)	No. of patients	Mean change (±SD)		
SPEED questionnaire score*	14	-3.21 ± 1.47	6	-1.50 ± 3.34	-1.71 (-5.36 to 1.94)	0.29
TBUT** (in seconds)	13	3.40 ± 4.06	6	2.73 ± 5.01	0.67 (-5.78 to 7.12)	0.85
MGS***	14	-1.14 ± 2.08	5	-0.90 ± 3.57	-0.24 (-4.37 to 3.89)	0.91
InflammaDry test****	12	-0.40 ± 0.48	5	-0.17 ± 0.22	-0.23 (-0.87 to 1.33)	0.33

**Figure 1:**
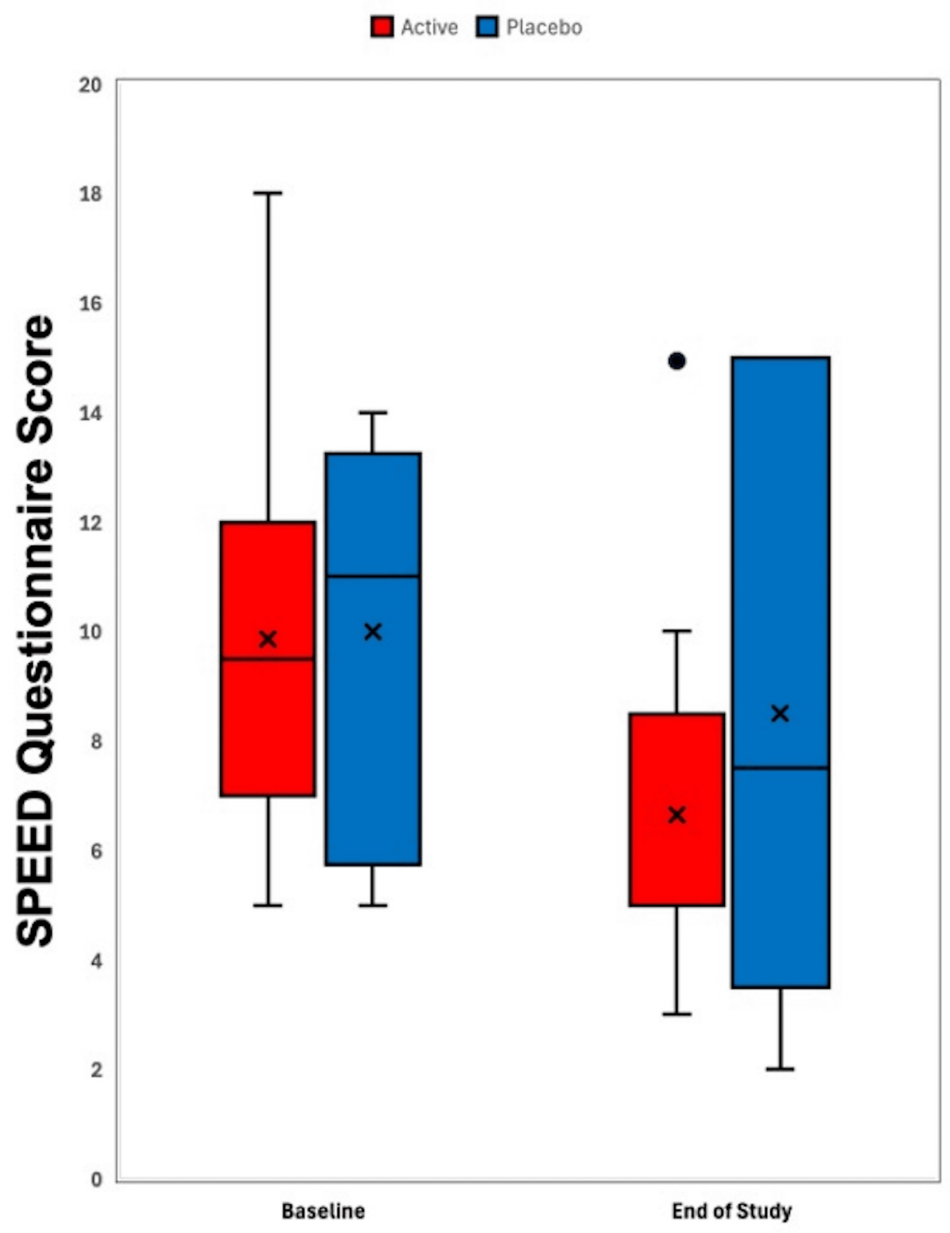
Box-and-whisker plot of SPEED questionnaire scores Box-and-whisker plot of SPEED questionnaire scores in the active supplement group (4,800 mg of sunflower lecithin per day) and placebo group (4,000 mg of olive oil per day) from baseline to 90 days. The boxes represent the 25th to 75th percentiles, with Xs indicating the mean and lines showing the median. Whiskers extend to the highest and lowest values within 1.5 times the interquartile range, and dots represent outliers. SPEED, Standard Patient Evaluation of Eye Dryness

**Figure 2:**
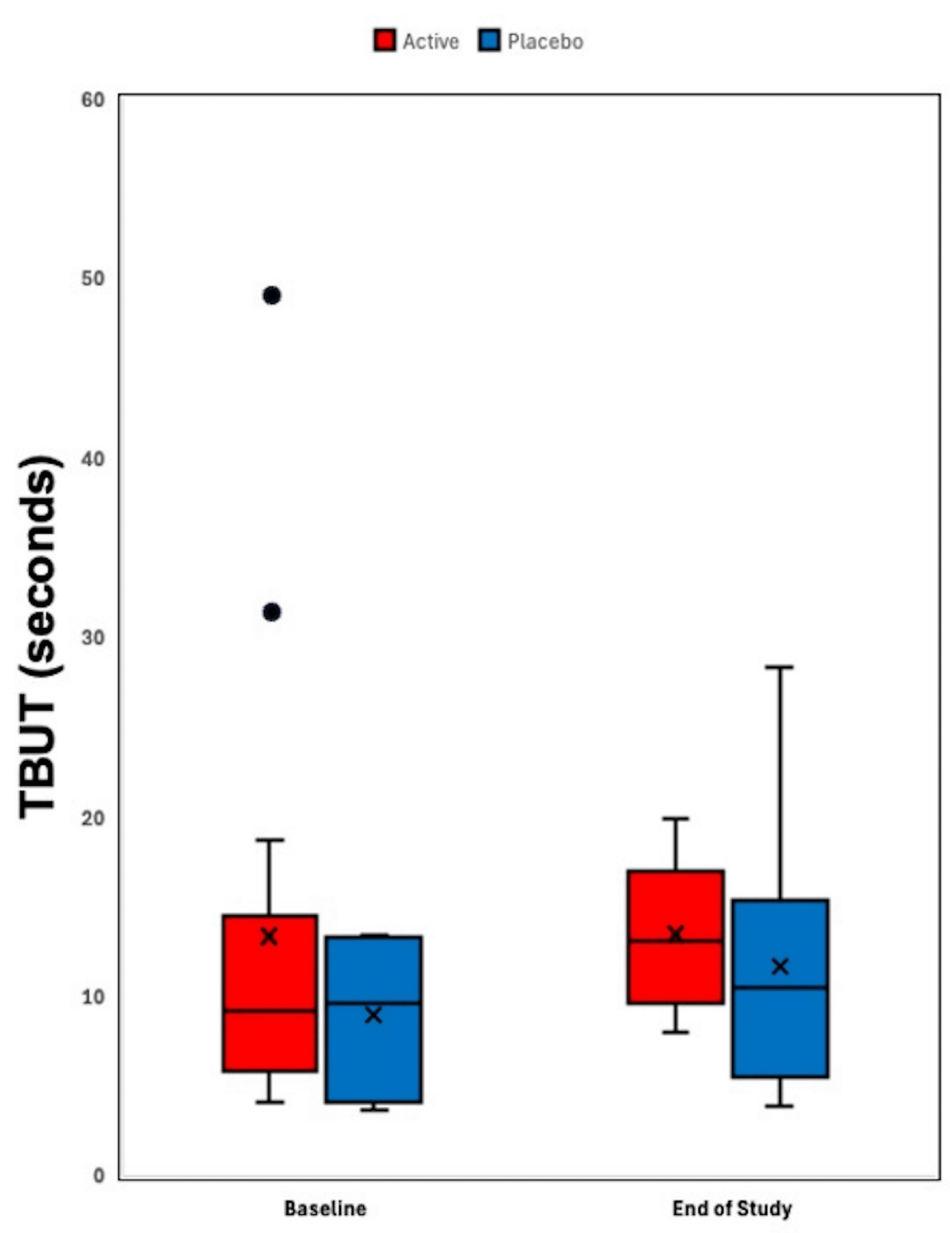
Box-and-whisker plot of TBUT Box-and-whisker plot of TBUT in seconds in the active supplement group (4,800 mg of sunflower lecithin per day) and placebo group (4,000 mg of olive oil per day) from baseline to 90 days. The boxes represent the 25th to 75th percentiles, with Xs indicating the mean and lines showing the median. Whiskers extend to the highest and lowest values within 1.5 times the interquartile range, and dots represent outliers. TBUT, tear film break-up time

**Figure 3:**
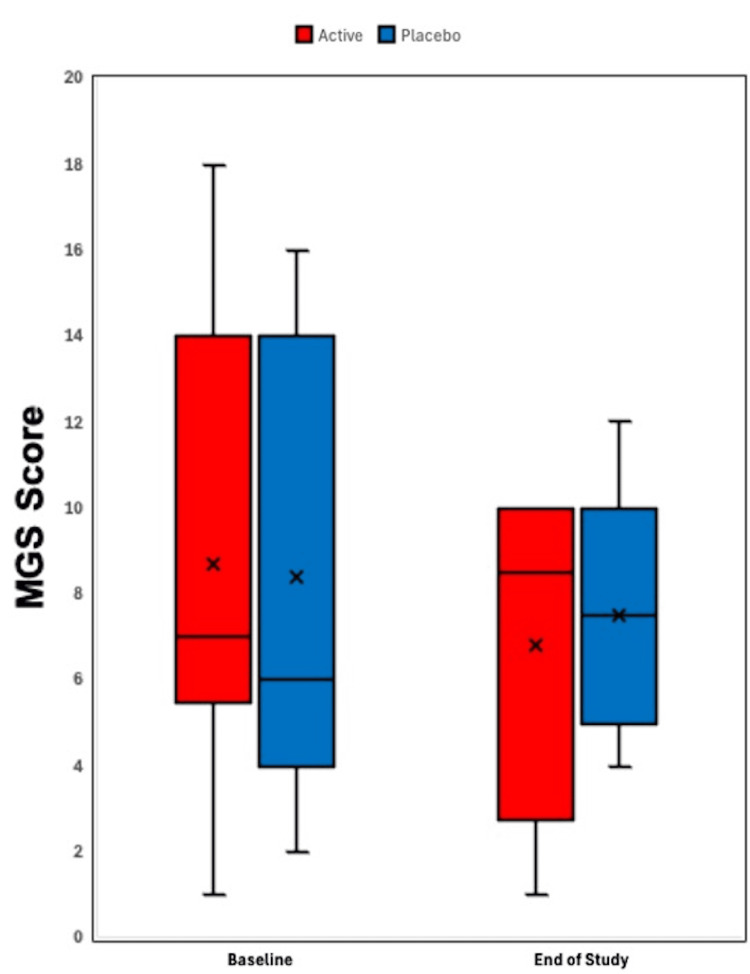
Box-and-whisker plot of MGS scores Box-and-whisker plot of MGS scores in the active supplement group (4,800 mg of sunflower lecithin per day) and placebo group (4,000 mg of olive oil per day) from baseline to 90 days. The boxes represent the 25th to 75th percentiles, with Xs indicating the mean and lines showing the median. Whiskers extend to the highest and lowest values within 1.5 times the interquartile range, and dots represent outliers. MGS, meibomian gland score

Figure 1 illustrates a box-and-whisker plot of SPEED questionnaire scores in the active supplement group (4,800 mg of sunflower lecithin per day) and placebo group (4,000 mg of olive oil per day) from baseline to 90 days. Figure 2 depicts a box-and-whisker plot of TBUT scores in the active supplement group (4,800 mg of sunflower lecithin per day) and placebo group (4,000 mg of olive oil per day) from baseline to 90 days. Figure 3 displays a box-and-whisker plot of MGS scores in the active supplement group (4,800 mg of sunflower lecithin per day) and placebo group (4,000 mg of olive oil per day) from baseline to 90 days.

## Discussion

In this study, we evaluated the impact of oral sunflower lecithin supplementation on meibomian gland function in adults with DES. While both the active sunflower lecithin group and the placebo group showed a trend towards improved dry eye symptoms as measured by the SPEED questionnaire (Figure 1), statistical significance was not present in either group. Similarly, no statistically significant improvement in the secondary outcomes, such as TBUT (Figure 2), MGS (Figure 3), and the InflammaDry immunoassay, was observed in either of the two study groups.

Similar to trials investigating dietary interventions for DES, such as the 2018 DREAM (Dry Eye Assessment and Management) trial, our study demonstrated a similar placebo effect [18]. In the DREAM trial, omega-3 supplementation failed to show a statistically significant improvement as compared to placebo, with both groups experiencing symptom relief over time. This substantial placebo effect is common in clinical trials involving subjective symptom assessments such as the SPEED questionnaire, where improvements are frequently noted regardless of the intervention. The Hawthorne effect may have manifested with an increased attention to ocular surface hygiene and follow-up, which could have contributed to symptomatic improvement independent of the assigned intervention. This effect is well-documented in chronic diseases that require patient-driven self-care and may have enhanced perceived symptom relief in both groups [23].

From a pathophysiology perspective, lecithin could enhance the tear film lipid layer by improving meibum fluidity in theory. Meibum is naturally composed of a complex mixture of nonpolar lipids with a small but functionally crucial phospholipid layer at the air-lipid interface [2]. Since phospholipids reduce surface tension and stabilize lipid interactions, supplementation with oral phospholipids has been proposed to improve meibomian gland secretion as well as quality [24,25]. However, in our study, oral sunflower lecithin did not demonstrate measurable benefits compared to a placebo, which could be attributed to limited systemic absorption or insufficient bioavailability of phosphatidylcholine at the level of the meibomian glands.

The TFOS DEWS III (Tear Film & Ocular Surface Society Dry Eye Workshop III) report emphasizes that DES is a multifactorial condition influenced by hormonal status, environmental exposures, systemic disease, patient behavior, and tear film biology [26]. These factors contribute to significant variability in clinical presentation, and further categorization of DES into aqueous-deficient dry eye and evaporative dry eye can better guide management and treatment regimens. TFOS DEWS III highlights that MGD is the most common cause of tear film instability, yet its severity and manifestations can vary widely between patients. This heterogeneity can create challenges in assessing treatment effects in smaller or shorter-duration trials such as ours. TFOS DEWS III also reinforces that tear film dynamics, lipid composition, and ocular surface inflammation interact in a complex feedback loop, which could explain our study's findings that lecithin did not significantly affect TBUT, MGS, or inflammatory markers. Overall, the updated framework provided by TFOS DEWS III supports the conclusion that oral sunflower lecithin may not exert a strong enough mechanistic influence on the lipid layer to overcome the biological variability and multifactorial drivers of DES identified in this updated consensus report.

Further insight can be drawn from recent therapeutic reviews that emphasize that successful management of DED often requires targeting multiple components of the tear film simultaneously [27]. Lipid-targeted therapies such as thermal pulsation, warm compresses, and lipid-based artificial tears act directly on the ocular surface and bypass systemic metabolism. In contrast, systemic agents like oral lecithin have more variable absorption and may not reach effective concentrations within the meibomian glands. Modern dry eye management, as described in TFOS DEWS III, focuses on precision medicine and identifying disease subtypes, such as evaporative dominant dry eye or obstructive versus hyposecretory MGD, to assist in guiding treatment selection. Since this study did not stratify patients by subtype, potential treatment effects in specific groups may have been diluted

One of the primary limitations of our study was the relatively small sample size, with only 20 patients in total completing the trial. Additionally, the predetermined duration of the study (90 days) may not have been long enough to detect more substantial improvements in meibomian gland function, especially given the chronic nature of DES, which may require longer durations of intervention to observe clinically meaningful changes. Another previously mentioned limitation of this study is the multifactorial nature of MGD and DES, which may lead to drastic day-to-day fluctuations of symptoms and tear-film composition. These factors include individual-related factors like eyelid hygiene and environmental factors like ambient temperature or humidity. The interaction between these factors and dietary supplements may have reduced the measurable impact of lecithin supplementation alone.

This study contributes one of the first placebo-controlled evaluations of oral sunflower-derived lecithin for MGD. The oral sunflower lecithin was well tolerated with no patient-reported adverse effects. Even though no statistically significant improvements were detected, these findings provide valuable groundwork for future research exploring higher doses, longer durations, combination therapy, or targeted trials in evaporative DED subtypes.

## Conclusions

The study’s randomized, double-masked design, which included rigorous assessment protocols for both objective measures (e.g., TBUT, MGS) and subjective measures (e.g., SPEED scores), enhances the reliability of the results. However, our findings do suggest that sunflower lecithin supplementation, as administered in this study, may not offer a clear therapeutic advantage over placebo in the management of MGD-related DED. Given the current lack of a gold standard treatment for MGD, there is a real unmet need for millions of patients suffering from DED. Further research is warranted to explore the role of phospholipids such as sunflower lecithin in the management of DED, possibly through larger studies with extended follow-up periods and consideration of additional factors like systemic inflammatory markers. Moreover, future studies might explore combination therapies, where lecithin is supplemented alongside other treatments, such as warm compresses or intense pulsed light therapy, to determine whether a multi-modal approach might yield better clinical outcomes.
